# Supplementary material for: Detection of central and obstructive sleep apneas in mice: A new surgical and recording protocol
Source: PLoS One. 2025 Mar 28;20(3):e0320650. doi: 10.1371/journal.pone.0320650 (PMC12048029; doi:10.1371/journal.pone.0320650)
Supplement: S1 File — (PDF) [file pone.0320650.s001.pdf]

Jul 31, 2024 Version 2

Detection of central and obstructive sleep apneas in mice: a new surgical and recording protocol V.2

DOI
dx.doi.org/10.17504/protocols.io.yxmvme54og3p/v2

Gabriele Matteoli[1], Sara Alvente[1], Chiara Berteotti[1], Dario Coraci[1], Viviana Lo Martire[1], Martina Lops[1], Elena Miglioranza[1], Alessandro Silvani[1], Emilia Volino[1], Giovanna Zoccoli[1], Stefano Bastianini*[1]

[1]Department of Biomedical and Neuromotor Sciences, Alma Mater Studiorum - University of Bologna, Bologna, Italy.

Stefano Bastianini*: (S.B.)

Stefano Bastianini
University of Bologna

OPEN ACCESS

DOI: dx.doi.org/10.17504/protocols.io.yxmvme54og3p/v2

Protocol Citation: Gabriele Matteoli, Sara Alvente, Chiara Berteotti, Dario Coraci, Viviana Lo Martire, Martina Lops, Elena Miglioranza, Alessandro Silvani, Emilia Volino, Giovanna Zoccoli, Stefano Bastianini* 2024. Detection of central and obstructive sleep apneas in mice: a new surgical and recording protocol. **protocols.io** https://dx.doi.org/10.17504/protocols.io.yxmvme54og3p/v2Version created by **Stefano Bastianini**

License: This is an open access protocol distributed under the terms of the **Creative Commons Attribution License**, which permits unrestricted use, distribution, and reproduction in any medium, provided the original author and source are credited

Protocol status: Working
We use this protocol and it's working

Created: July 22, 2024

Last Modified: July 31, 2024

Protocol Integer ID: 104300

Keywords: Mouse, Central sleep apnea, Obstructive sleep apnea, Sleep, Breathing, Whole-body plethysmography

Funders Acknowledgement:
This work was co-funded by the European Union (NextGenerationEU) under the National Recovery and Resilience Plan (PNRR) within the framework of the project "ESTROSA: Energy-autonomous System for TReatment of Obstructive Sleep Apnea"
Grant ID: Code MUR P2022RTCCA_001 – CUP J53D23014050001
This work was co-funded by by the Italian Ministry of Health with the grant PRIN2022 for the project "CORSA: Chemogenetic and Optogenetic Rescue of Sleep Apnea in mice"
Grant ID: Code MUR 2022CR32TM_001 – CUP J53D2301094000

Abstract

Sleep apnea is a common disorder in humans and consists of recurrent episodes of cessation of breathing or decrease in airflow during sleep. Sleep apneas can be classified as central (CSA) or obstructive (OSA), based on their origin. CSA results from impairments of the inspiratory drive generated by the brainstem with a consequent interruption of diaphragm muscle effort, while OSA occurs due to upper airway obstruction during inspiration with a continued or increased respiratory effort. Mice are valuable tools to expedite the comprehension of the pathophysiology and genetics of sleep apneas and the development of targeted therapies to contrast sleep-disordered breathing and its adverse consequences on health. Therefore, we provide a method for distinguishing between CSA-like and OSA-like events during sleep in mice by simultaneously recording sleep states, breathing pattern, and diaphragm electromyographic activity inside a whole-body plethysmograph.

We applied this technique in adult C57BL/6J wild-type mice and in a mouse model of Down syndrome (PMID: 34509609).

Materials

Step 1: Preparation of laboratory-made electrodes

- Multi-stranded stainless-steel wires with an insulating coating. We currently use wire model #793500 (0.002-inches diameter, 25-feet length) produced by A-M Systems LLC (WA, USA; link: https://www.a-msystems.com).
- Working tools (scissors, tweezers, forceps, clamps, coating remover).
- Zinc chloride-based flux paste or solution.
- Soldering equipment.
- Surgical microscope.
- Stainless-steel screws. We currently use screws model 00-96x3/32 (2.4-mm length and 1.2-mm diameter) produced by Protech International, Inc. (VA, USA; link: https://protechinternational.com).
- Electrical connectors with two terminal pins. We currently use connectors model 701-9925 (0.5-cm wide and 0.3-cm high) sold by RS Components s.r.l. (Milan, Italy; link: https://it.rs-online.com/web).
- Hot air flux generator.
- Biocompatible acrylic paste. We currently use paste DuraLay produced by Reliance Dental Manufacturing LLC (IL, USA; link: https://www.reliancedental.net).
- Electrical multimeter.

Step 2: Sterilization of surgical materials

- Autoclave.
- Alcohol-based disinfectant solutions. We currently use LH Benzalcol Ferri (benzalkonium chloride 0.30 g and ethyl alcohol 71%) produced by Lombarda H s.r.l. (Milan, Italy; link: https://www.lombardah.com).
- UV lamp.

Step 3: Surgical implantation of electrodes in mice

- Isoflurane.
- Analgesic therapy (0.1 mg of Carprofen 50 mg/mL, Rimadyl®, Zoetis, Rome, Italy); subcutaneous injection of 200 μL of a solution of 10 μL of Rimadyl® dissolved in 1 mL of saline).
- Antibiotic therapy (3’750 I.U. of 250’000 I.U./mL benzathine benzylpenicillin and 1.5 mg of 100 mg/mL dihydrostreptomycin sulfate, Strong Rubrocillin®, MSD Animal Health s.r.l., Milan, Italy); subcutaneous injection of 15 μL of Strong Rubrocillin® dissolved in 800 μL of saline.
- Thermoregulated heating pad produced by Harvard Apparatus (MA, USA; link: https://www.harvardapparatus.com).
- Closed chamber for the induction of anesthesia. We currently use a chamber with a sliding top lid equipped with inlet and outlet tubes for the transport of anesthetic gas (24x12x18 cm).
- Shaving cream and razors.
- Iodine solution. We currently use 7.5% polyvinylpyrrolidone iodine produced by Lombarda H s.r.l. (Milan, Italy; link: https://www.lombardah.com).
- Inclined plane. We currently use a 20x20 cm platform with an inclination of 20°.

- Surgical tools (forceps, tweezers, scissors with a curved tip, mosquito clamps, microsurgical scissors, periosteum remover, needle holder).
- Gauzes.
- Surgical microscope.
- Non-absorbable surgical threads (silk 5/0 and polyester 3/0).
- Stereotaxic apparatus. We currently use model 51600 Lab Standard™ Stereotaxic Instruments produced by Stoelting Co (IL, USA; link: https://stoeltingco.com).
- Hydrogen peroxide (3% H_2O_2).
- Surgical microdrill.
- Screwdriver for surgical screws.
- Cement for dental use. We currently use 3M™ ESPE™ RelyX™ Unicem produced by 3M (Milan, Italy; link: https://www.3mitalia.it).
- Curved needle (1/2 curved round, bodied size 16, 18-mm chord length)
- Biocompatible acrylic paste. We currently use paste DuraLay produced by Reliance Dental Manufacturing LLC (IL, USA; link: https://www.reliancedental.net).
- Electrical multimeter.

Step 4: Recording within a whole-body plethysmograph

- Isoflurane.
- Lightweight cable.
- Whole-body plethysmography chamber. We currently use chamber model PLY4223 Buxco® produced by Data Sciences International (DSI) (NC, USA; link: https://www.datasci.com). This apparatus was modified by inserting a solid, machined 10-cm diameter Plexiglas block to reduce its internal volume to 0.97 L and equipping it with a rotating electrical commutator (see below) to prevent twisting of the mouse wire tether during the recordings.
- Breathable air cylinders for air inlet into the WBP chamber.
- Rotating electrical commutator. We currently use model SL6C/SB produced by Protech International, Inc. (VA, USA; link: https://protechinternational.com).
- High-precision differential pressure transducer. We currently use model DP103–06 coupled to a CD223 digital transducer indicator produced by Validyne Engineering (CA, USA; link: https://www.validyne.com).
- Micro-syringe for WBP chamber calibration. We currently use a 100-µL micro-syringe produced by Hamilton Company (CA, USA; link: https://www.hamiltoncompany.com).
- Data acquisition system. We currently use a PCI6224 digital acquisition board produced by Emerson (former National Instruments, TX, USA; link: https://www.ni.com) and operated with software written in LabVIEW.
- Thermometer/hygrometer probes for measuring the temperature and the humidity of the chamber. We currently use the PC52–4-SX-T3 sensor produced by Michell Instruments (former Rense Instruments, UK; link: www.michell.com).

Step 5-6: Discrimination of wake-sleep states in mice and sleep apnea detection and categorization into CSA and OSA

- We use analysis software written in MATLAB (The MathWorks Inc., MA, USA), including an implementation of a validated semi-automatic algorithm for sleep scoring (SCOPRISM)(2).

CITATION

Bastianini S, Berteotti C, Gabrielli A, Del Vecchio F, Amici R, Alexandre C, Scammell TE, Gazea M, Kimura M, Lo Martire V, Silvani A, Zoccoli G (2014). SCOPRISM: a new algorithm for automatic sleep scoring in mice..

LINK

https://doi.org/10.1016/j.jneumeth.2014.07.018

Overview

1 Figure 1 summarizes the chronological steps of the protocol.

Figure 1. Overview of the protocol

- The experimental protocol involves the crafting (Day -1) and the surgical implantation (Day 0) of the electrodes for the recording of electroencephalogram (EEG), nuchal muscle electromyogram (nEMG), and diaphragm electromyogram (DIA).
- After an appropriate period of full post-operatory recovery (Day 5), mice underwent a 7-hour session inside a whole-body plethysmograph to simultaneously record EEG, nEMG, DIA, and ventilation (VENT).
- In the analysis phase, EEG and nEMG signals are used for the sleep scoring, while VENT and DIA signals are employed for the classification of sleep apneas.

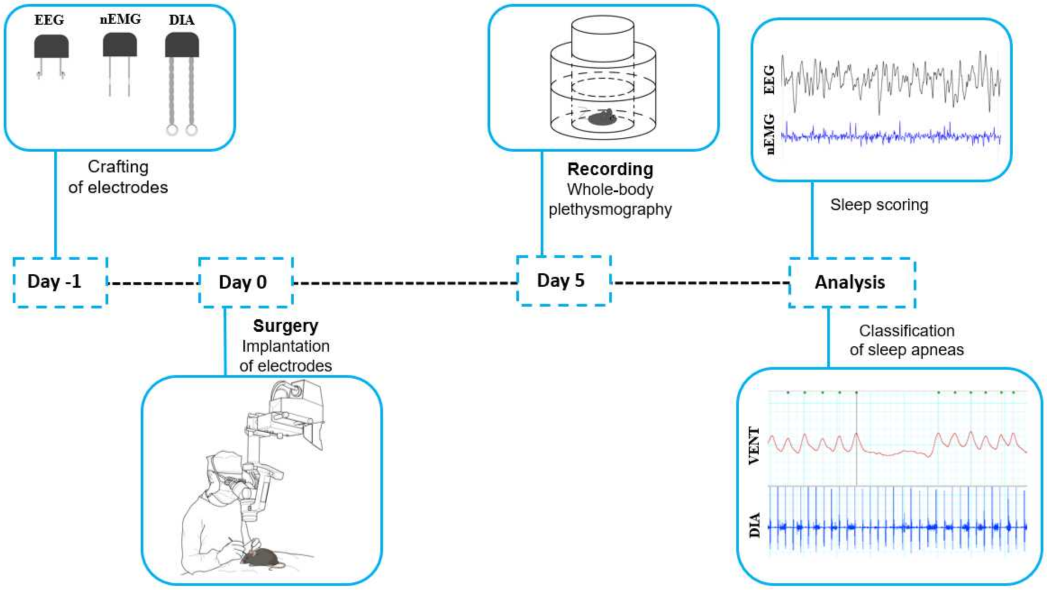

Step 1: Crafting of electrodes

2 In this section, we describe how to craft the electrodes for electroencephalogram (EEG), nuchal muscle electromyogram (nEMG), and diaphragm electromyogram (DIA) recordings.

Note

Timing: 00:10:00 - 00:20:00

3 **Preparation of EEG electrode** (Figures 2A-3A and Video 1)

3.1 Cut a pair of 1.2-cm long multi-stranded stainless-steel wires coated with an insulating sheath.

3.2 Gently remove 2-3 mm of the coating from both ends of each wire.

3.3 Solder one end of each wire to a stainless-steel screw; we suggest using screws with a length of 2.4 mm and a diameter of 1.2 mm.

3.4 Solder the uninsulated remaining end of each wire to a connector equipped with two terminal pins (one wire per pin); we suggest using connectors about 0.5 cm wide and 0.3 cm high.

4 **Preparation of nEMG electrode** (Figures 2B-3B and Video 2)

4.1 Cut a pair of 4-cm long multi-stranded stainless-steel wires coated with an insulating sheath.

4.2 Remove 2-3 mm of the coating from one end of each wire and at 1.5 cm from it.

4.3 Solder the uninsulated end of the wire to a connector equipped with two terminal pins (one wire per pin).

5 **Preparation of DIA electrode** (Figures 2C-3C and Video 3)

5.1 Cut a pair of 12-cm long multi-stranded stainless-steel wires coated with an insulating sheath.

5.2 Bend each wire into a hairpin-like shape.

5.3 Twist the wire on itself until it reaches a final length of 6 cm, ensuring the hairpin bend is enlarged and shaped as a circle with about 2 mm in diameter.

5.4 Gently melt the coating along the wire using a hot air flux to stabilize the shape of the electrode.

5.5 Remove 2-3 mm of the coating from the free end of each wire and the circular hairpin bend.

5.6 Solder the uninsulated end of each wire to a connector equipped with two terminal pins (one wire per pin).

6 **Figure 2. Schematic representation of the electrodes**

- Schematic representation of electrodes for electroencephalogram (EEG, panel A), nuchal muscle electromyogram (nEMG, panel B), and diaphragm electromyogram (DIA, panel C) recordings.

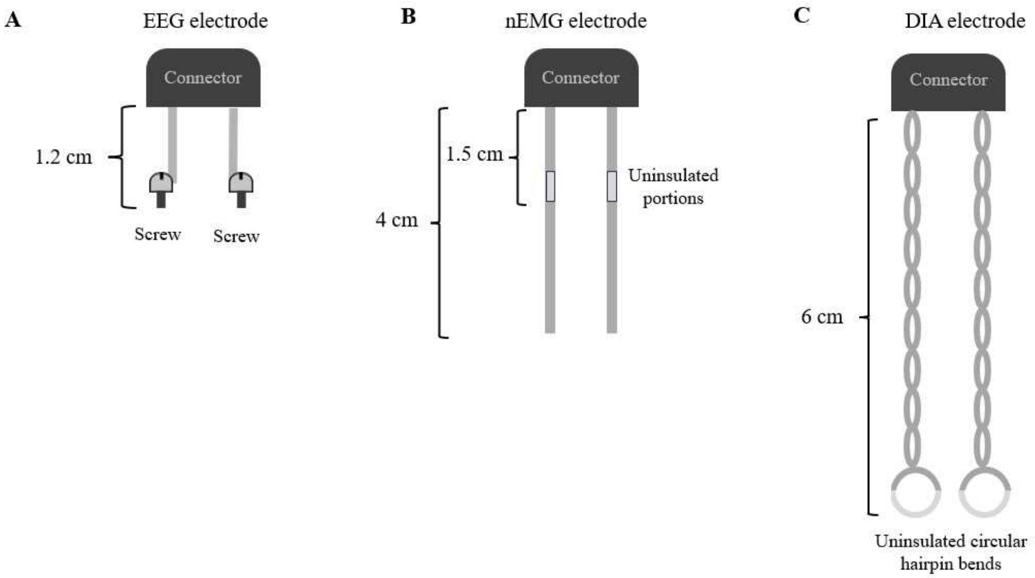

Figure 3. Photographs of the electrodes

- Photographs of electrodes for electroencephalogram (EEG, panel A), nuchal muscle electromyogram (nEMG, panel B), and diaphragm electromyogram (DIA, panel C) recordings.

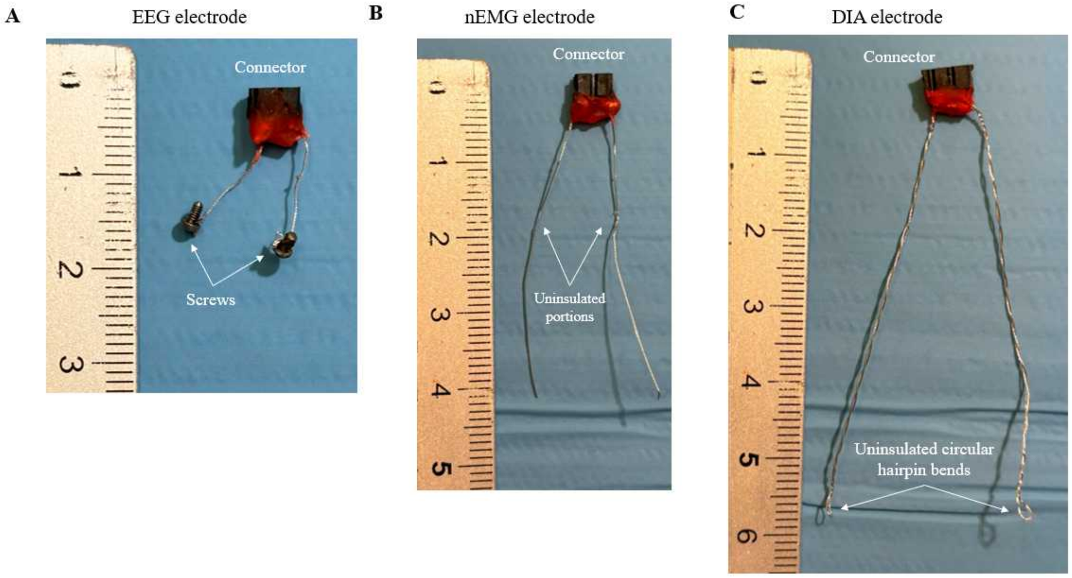

Video 1. Crafting of the electroencephalogram electrode

- Preparation of the electroencephalogram (EEG) electrode.

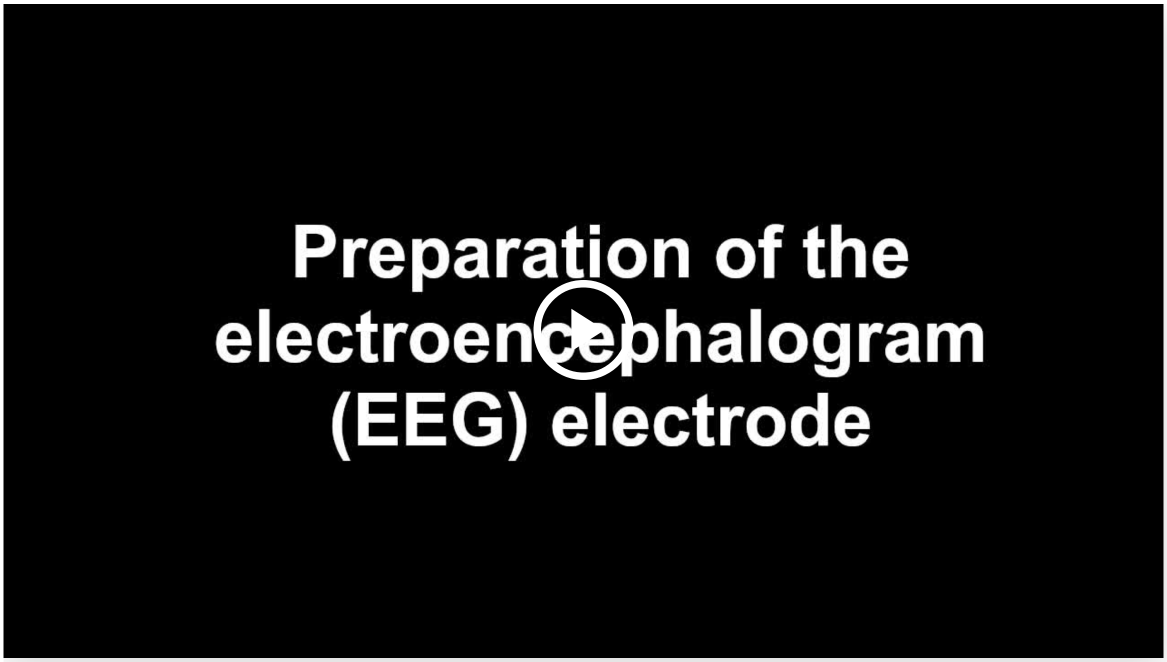

Video 2. Crafting of the nuchal muscle electromyogram electrode

- Preparation of the nuchal muscle electromyogram (nEMG) electrode.

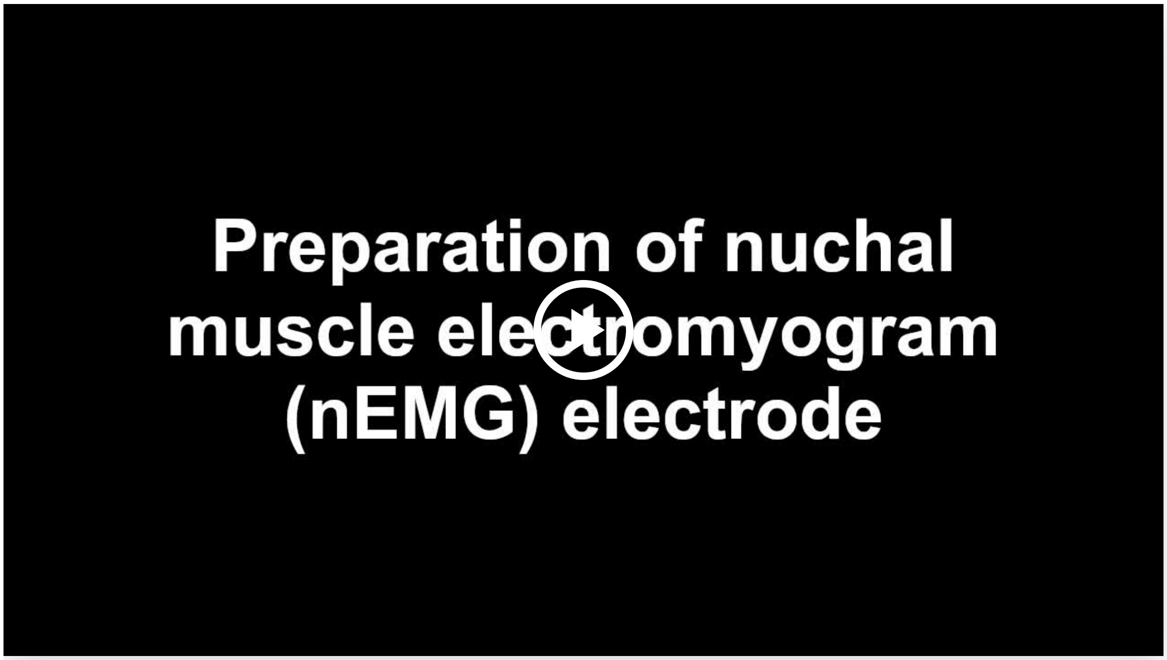

Video 3. Crafting of the diaphragm electromyogram electrode

- Preparation of the diaphragm electromyogram (DIA) electrode.

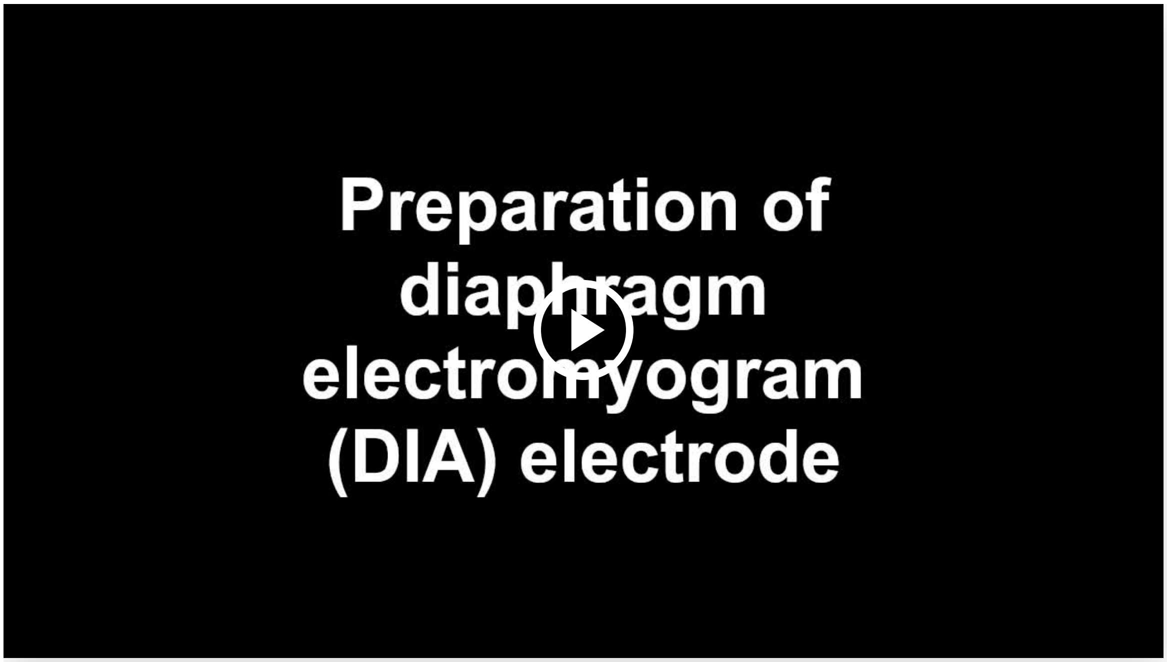

7

Note

Tip:

- The dimensions of all electrodes can be adapted to the animal's size (e.g., shorter wires may be required for younger and smaller mice).

8

Note

Critical points:

- For a longer and more stable duration of the soldering, we suggest using a commercial catalyst (e.g., zinc chloride-based solution).
- To safeguard the electrodes from potential damage during the removal of the coating, we recommend performing this procedure under a surgical microscope (this step is strongly recommended for the DIA electrode).
- To increase electrode longevity, we suggest applying an acrylic paste to cover the soldering.
- For validation of electrode functionality, we recommend testing the electrical conductance of the uninsulated portions using a suitable tool (e.g., a multimeter tester) before using it. We suggest measuring the electrical resistance using a threshold of 20,000 ohms.

Step 2: Sterilization of surgical materials

15m

9 Surgery is performed under aseptic conditions.
Surgical instruments (forceps, tweezers, scissors with a curved tip, mosquito clamps, microsurgical scissors, periosteum remover, and needle holder) must be sterilized by an autoclave cycle at 121 °C for 00:15:00 . Alternatively, they can be disinfected with specific solutions (e.g., 0.35% peracetic acid solutions).

15m

Step 3: Surgical implantation of electrodes in mice

10 The procedure is performed under aseptic conditions with the mouse anesthetized (isoflurane 2-2.5% in pure oxygen).
Analgesic and antibiotic therapy are administered subcutaneously at the beginning and at the end of the surgery, respectively. During the entire duration of the surgery, the mouse is placed over a thermoregulated heating pad to prevent hypothermia.

Note

Timing: about 01:15:00

11 **Anesthesia and surgical field disinfection**

11.1 Place the mouse in a closed chamber to induce the anesthesia.

11.2 Place the mouse on the surgical table, ensuring that its nose and mouth are located within the anesthesia tube.

11.3 Shave the mouse in the subcostal area (under the last rib) and the skin above the head between the ears (Figure 4).

11.4 Disinfect the shaved surgical fields with an iodine solution.

12 **Figure 4. Surgical fields**

- Photographs of the surgical fields after shaving showing the subcostal area (under the last rib, panel A) and the skin above the head between the ears (panel B).

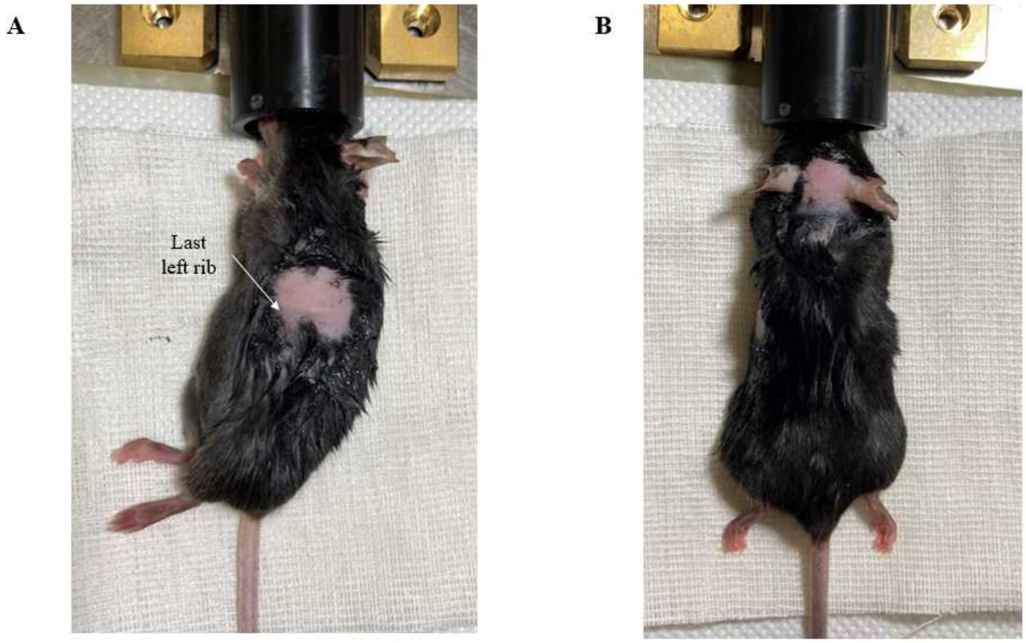

13 **DIA electrode implantation**

13.1 Place the mouse on an inclined plane with the head at a lower level than the rest of the body in order to obtain a better exposure of the surgical field.

13.2 Use scissors to make a 0.5-cm vertical skin incision over the skull between the ears (Figure 5A and Video 4).

13.3 Make a second 0.5-cm vertical skin incision on the flank just below the last rib (Figure 5B and Video 4).

13.4 Using surgical scissors with curved tips, create a subcutaneous tunnel connecting the two incisions (Video 5).

13.5 Pass the DIA electrode subcutaneously from the head to the flank incision with the help of surgical forceps and a mosquito clamp. The electrode connector must remain on the animal's head (Figure 6 and Video 5).

13.6 Place the mouse on the right flank.

13.7 With the help of a surgical microscope, make a 0.5-cm horizontal cut in the abdominal muscle wall and the peritoneum under the last rib using microsurgical scissors (Figure 7A).

13.8 Identify the last rib attached to the sternum and the eighth intercostal space (i.e., the space between the two last ribs connected to the sternum) (Figure 7B).

13.9 Position the DIA electrode in the correct position (Figure 8). Video 6 provides a detailed step-by-step guide for implanting the DIA electrode, illustrating the following process:

1. Pass a thin non-absorbable surgical thread through the eight intercostal space from outside to inside the abdominal cavity.
2. Bend the DIA electrode wires to adapt to the natural shape of the abdominal cavity of the mouse.
3. Pass the surgical thread through one of the circular hairpin bends of the DIA electrode.
4. Pass the surgical thread back through the same intercostal space in the peritoneal wall, this time proceeding from intrabdominal to external.
5. Cut the surgical thread, making sure to leave enough length to tie a knot.
6. Insert the DIA electrode's circular hairpin bend into the abdominal cavity.
7. Pull the suture thread until the circular hairpin bend of the DIA electrode is in contact with the abdominal surface of the diaphragmatic muscle and tie a knot.

13.10 Repeat the same procedure for the second DIA electrode's circular hairpin bend, ensuring that the uninsulated circular portions of the electrode are positioned close to each other, but avoiding any contact between them (Video 6).

13.11 Apply suture stitches to close the abdominal muscles around the electrode wires. A second series of stitches must be applied to the flank skin incision (Figure 9).

14 **Figure 5. Skin incisions**

- Photographs of the vertical incision over the skull between the ears (panel A) and on the flank just below the last rib (panel B).

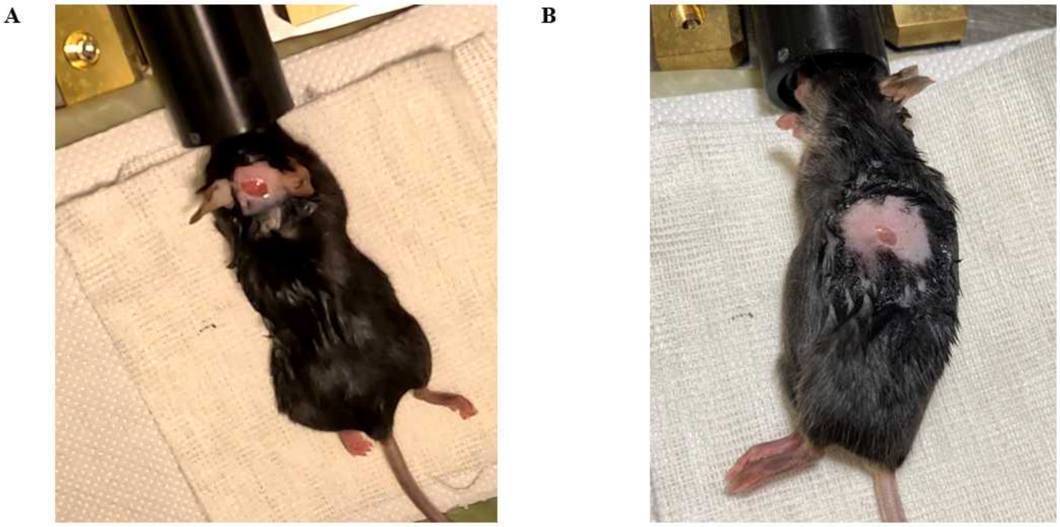

Figure 6. Insertion of the diaphragm electrode

- Photographs of the top (panel A) and side (panel B) view of the mouse with the diaphragmatic electrode inserted in the subcutaneous space.

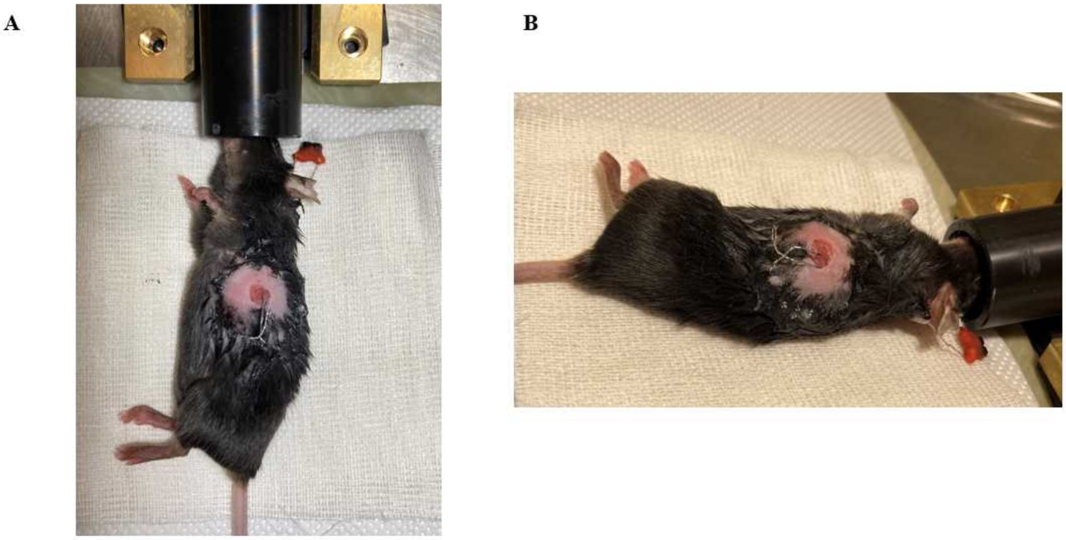

Figure 7. Identification of the last rib

- Panel A: photograph of the incision in the abdominal muscle wall and the peritoneum under the last rib.

- Panel B: magnification of the last rib attached to the sternum (black arrows) and the eighth intercostal space (VIII).

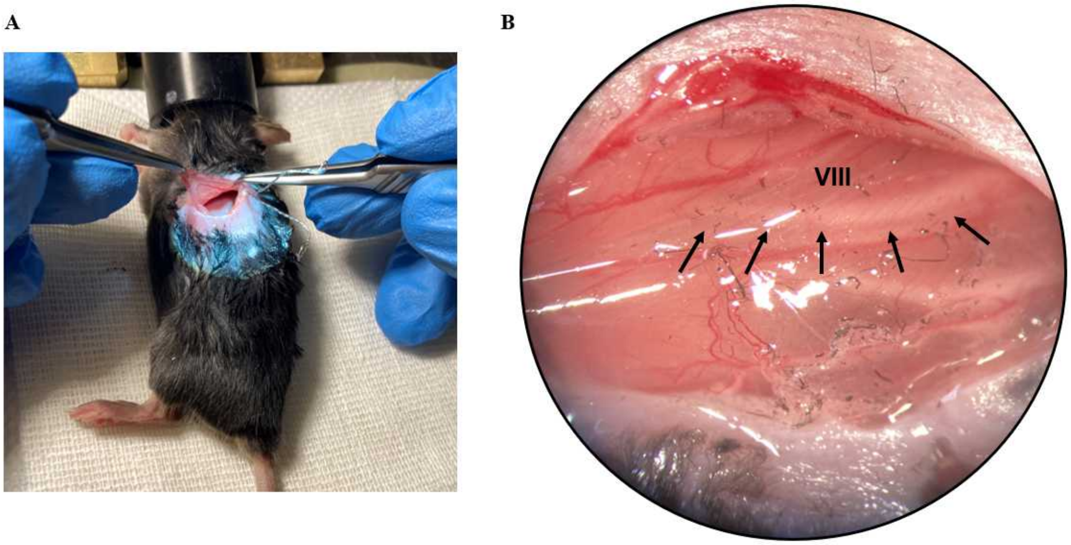

Figure 8. Schematic representations of the diaphragmatic electrode positioning

- Circular uninsulated hairpin bends of the diaphragmatic (DIA) electrode were sutured to the muscles of the peritoneal cavity in the eighth intercostal space, so that the DIA electrode was in contact with the abdominal surface of the diaphragm (orange curved lines).

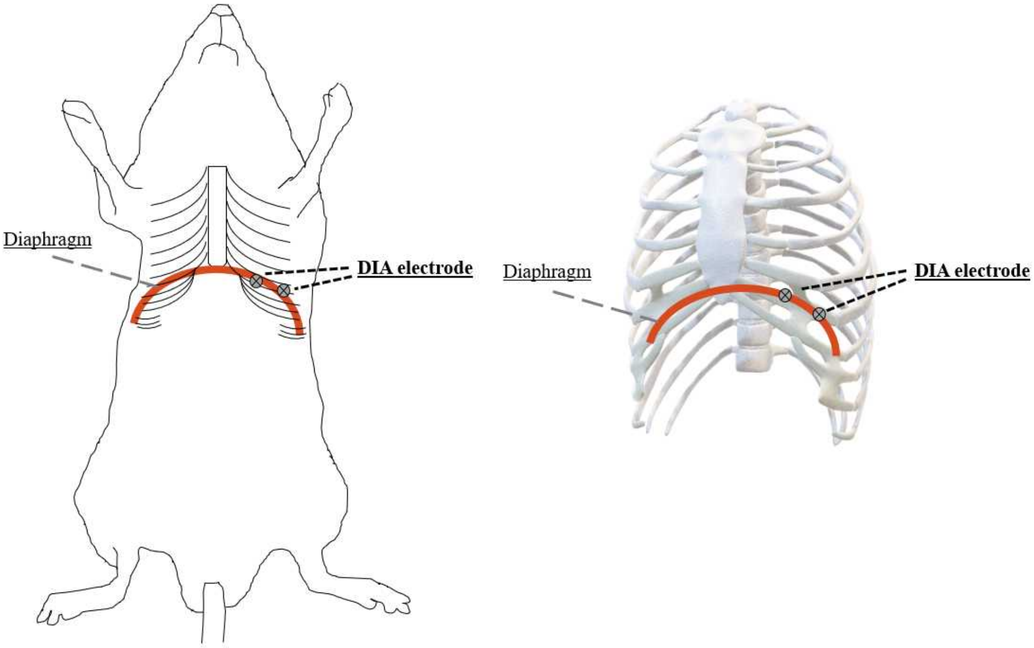

Figure 9. Surgical sutures

- Photographs of the suture stitches applied to close the abdominal muscles around the diaphragm electrode wires (panel A) and the flank skin incision (panel B).

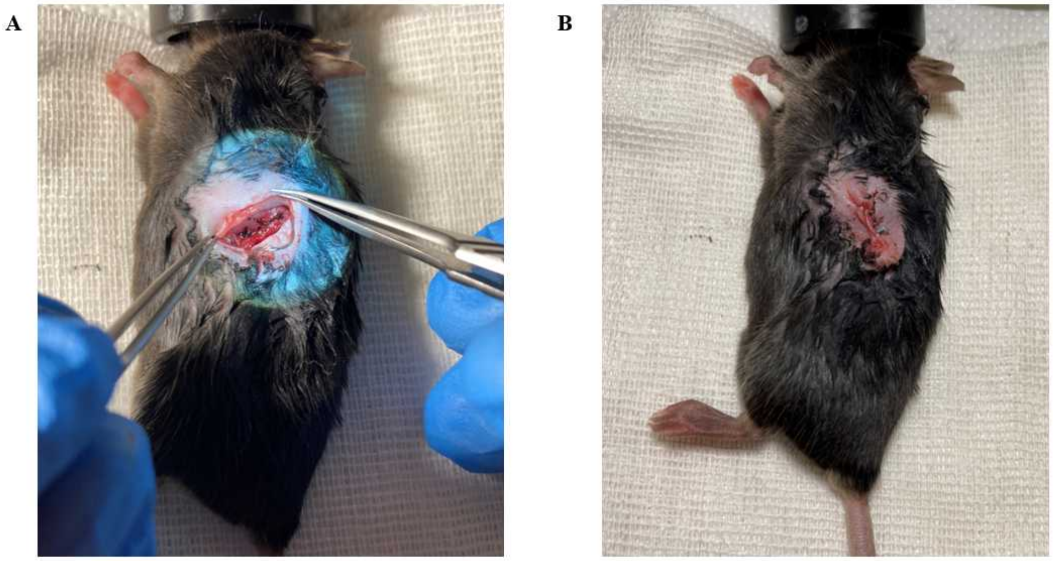

Video 4. Skin incisions

- Execution of the skin incisions over the skull between the ears and on the flank just below the last rib.

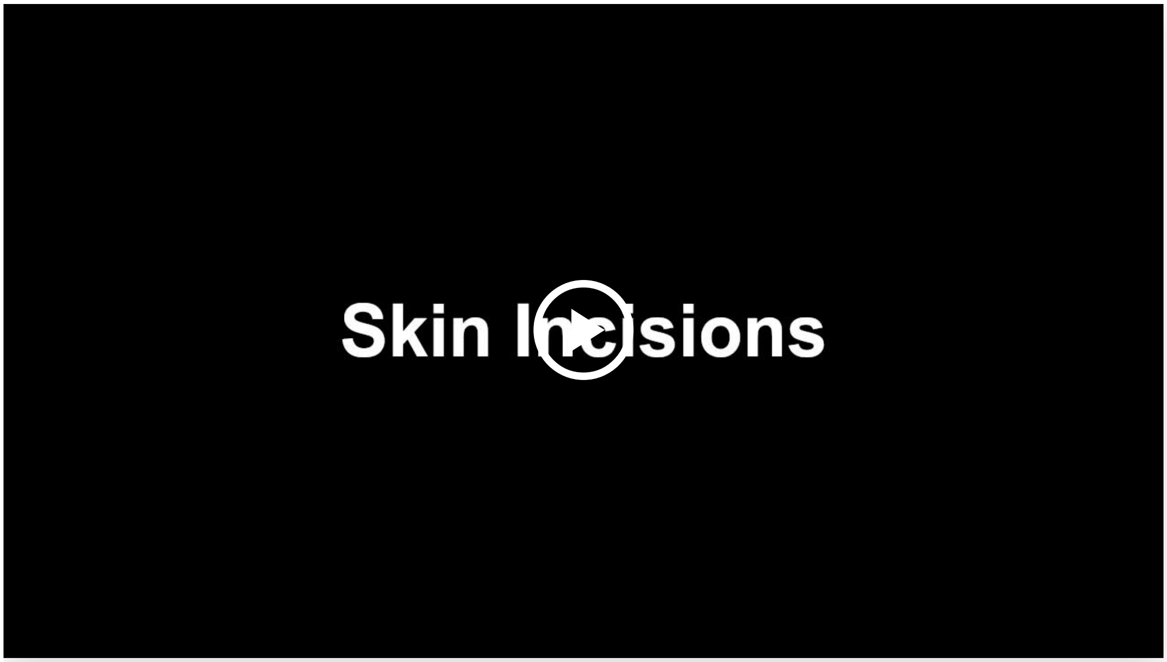

Video 5. Insertion of the diaphragm electromyogram electrode

Insertion of the diaphragm electromyogram (DIA) electrode in the subcutaneous space between the skin incisions on the head and the flank.

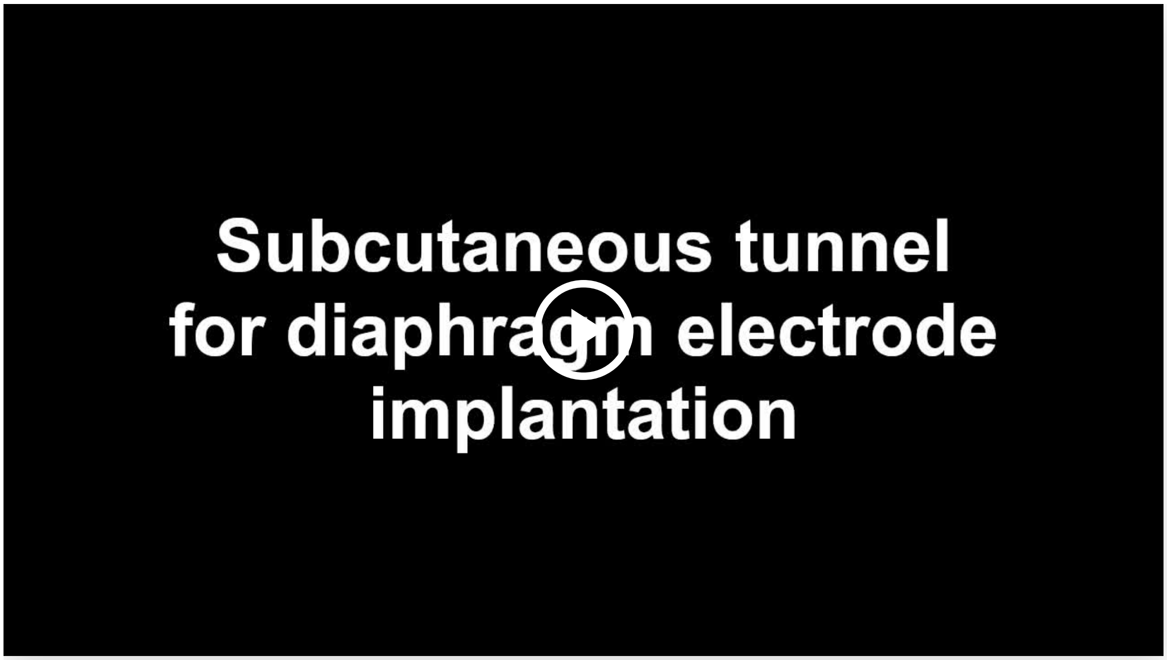

Video 6. Implantation of the diaphragm electromyogram electrode

- Detailed step-by-step guide for implanting the diaphragm electromyogram (DIA) electrode.

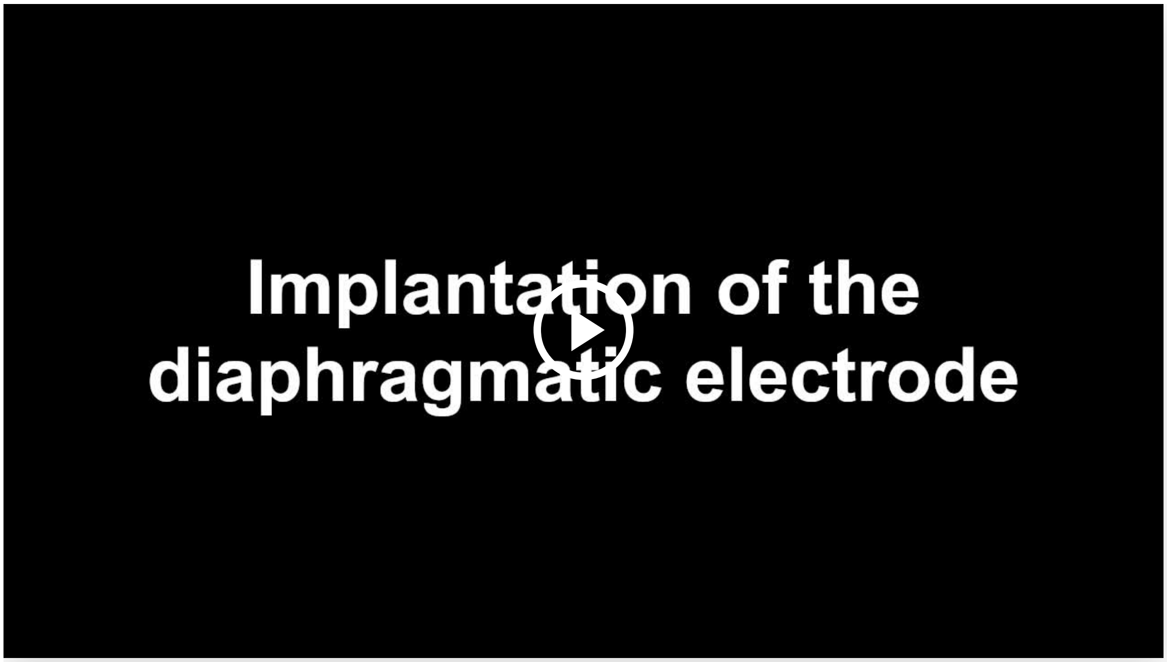

15 **EEG electrode implantation**

15.1 Place the mouse in a prone position on the stereotaxic apparatus to immobilize and expose the skull (Figure 10).

15.2 Remove the periosteum from the skull and locally apply a drop of hydrogen peroxide (3% H_2O_2) to remove debris and better highlight the cranial sutures.

15.3 Identify bregma and lambda (i.e., the anterior and posterior crossing points of cranial sutures) (Figure 11A and B).

15.4 Using a surgical microdrill, make four holes in the frontal and parietal bones 1 mm anterior and 1 mm lateral to both bregma and lambda (Figures 11C and 12A).

15.5 Insert two stainless-steel screws in the holes on one side of the skull in contact with the dura mater for better anchoring and balancing the head cap structure to the skull (Figure 12B).

15.6 Securely fasten the screws by turning them clockwise (usually one complete turn is sufficient; pay attention not to screw them excessively, which could damage the underlying cerebral cortex).

15.7 Insert the two screws of the EEG electrode in the two remaining contralateral holes of the skull in contact with the dura mater (Figure 12C).

15.8 Joint the screws and the connector of the EEG electrode to the skull bone using cement for dental use (e.g., biocompatible and atoxic resins composed of methacrylate and apatite).

16 **Figure 10. Stereotaxic apparatus**

- Photograph of an immobilized and exposed mouse skull.

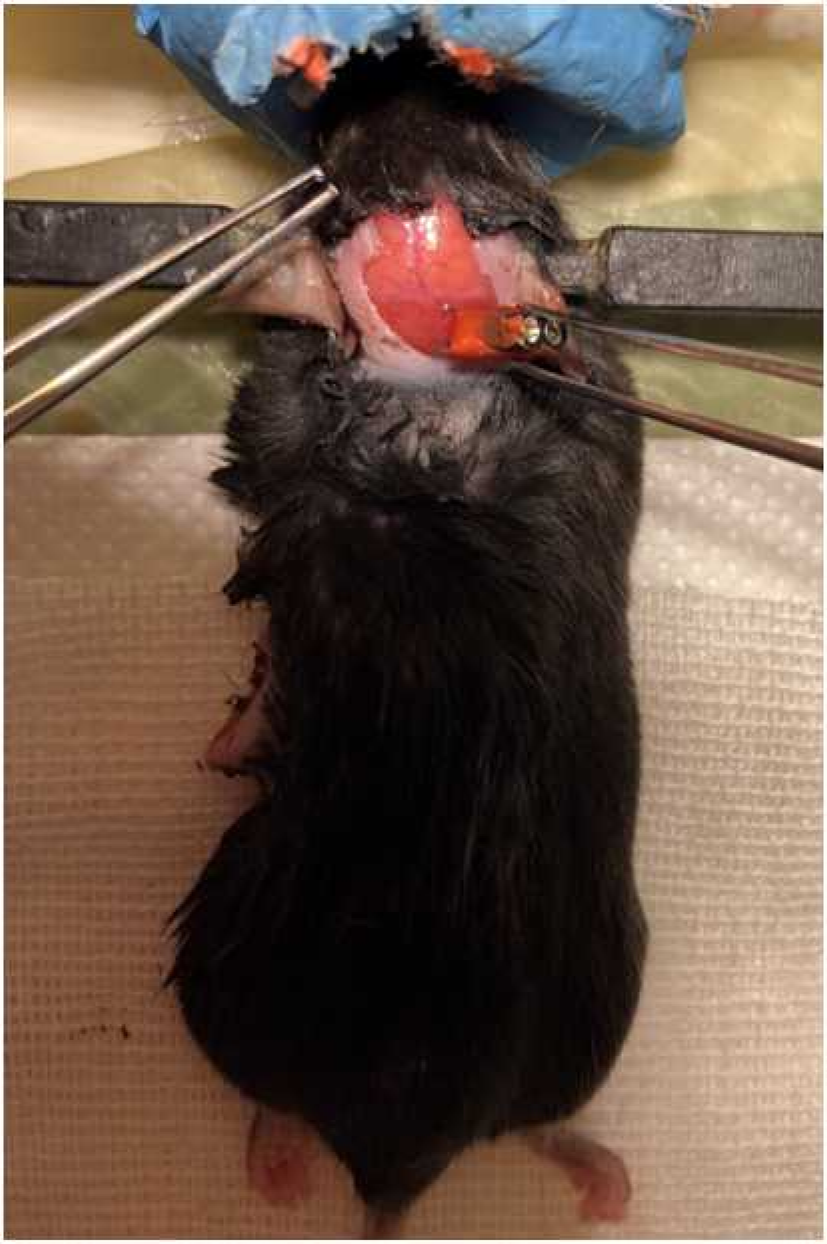

Figure 11. Mouse's head and screws positioning

- Panel A. Photograph of a mouse skull with bregma and lambda (anterior and posterior crossing points of cranial sutures, respectively) marked after the application of hydrogen peroxide.
- Panel B: Magnifications of a mouse skull before (left) and after (right) hydrogen peroxide application.
- Panel C: Schematic representation of the mouse head and screws positioning. The four holes were made 1 mm anterior and 1 mm lateral to bregma and lambda on both side on the frontal and parietal bones. Electroencephalographic (EEG) electrode screws were

inserted in the holes to the right, while the anchoring screws were inserted in the holes to the left.

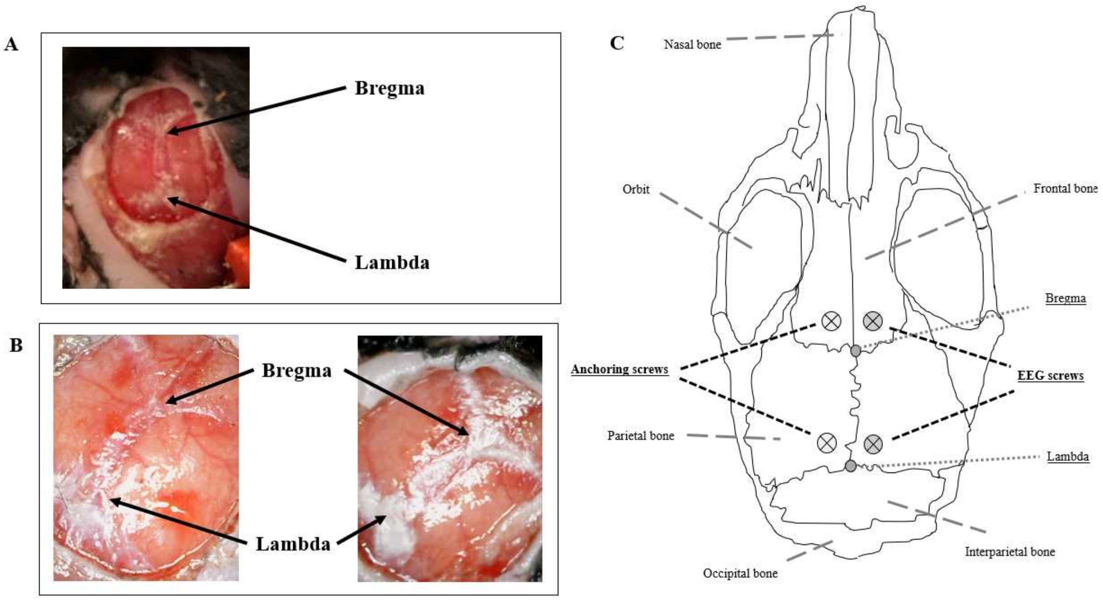

Figure 12. Implantation of the electroencephalographic electrode

- Panel A: photograph (left) and magnification (right) of four holes in the frontal and parietal bones performed using a surgical microdrill.
- Panel B: insertion of the two anchoring stainless-steel screws in the holes on the left side of the skull.
- Panel C: insertion of the screws of the electroencephalographic electrode in the holes on the right side of the skull.

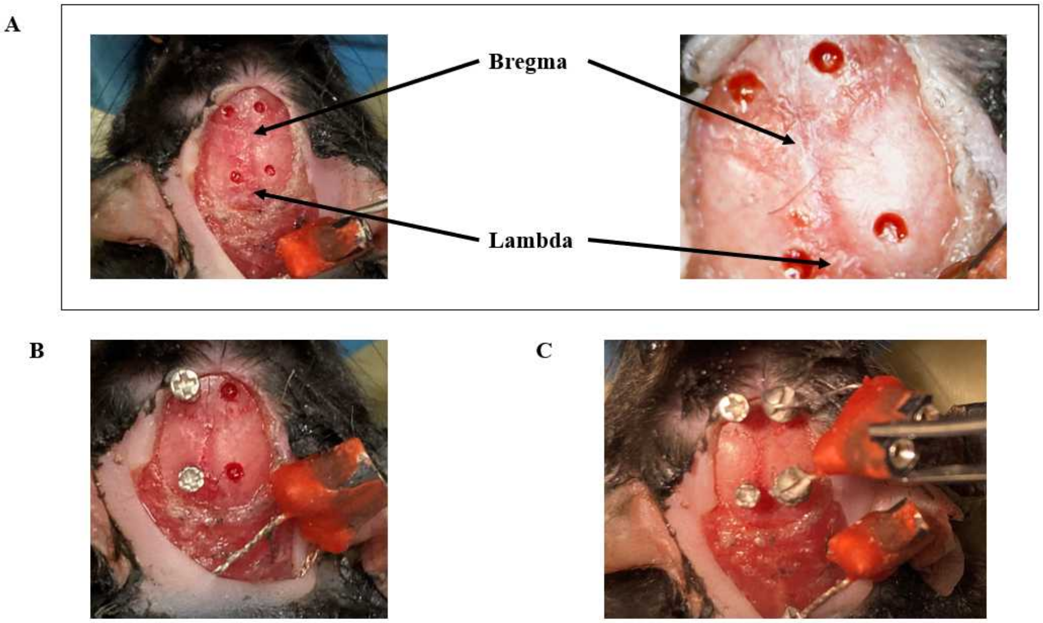

17 **Implantation of the nEMG electrode**

17.1 Insert the nEMG electrode wires bilaterally in the nuchal muscles using a curved needle, ensuring that the uninsulated portion of the wire is located inside the muscle (Figure 13 A and B).

17.2 Block the wires with a knot to keep them in position (Figure 13C).

17.3 Incorporate the connectors of the EEG, nEMG, and DIA electrodes into a biocompatible acrylic paste for dental use on the mouse's skull (Figure 14).

18 The implantation of the EEG and nEMG electrodes is also explained in detail in Video 7.

Note

Critical points:

- Respiratory depression due to anesthesia must be continuously monitored during the entire procedure based on changes in the animal's breathing rate and tidal volume, and the anesthetic fraction in the inspired gas flow must be adjusted accordingly, taking account of the depth of anesthesia.
- The implantation of electrodes requires basic surgical skills.
- Ensure accurate identification of the last rib attached to the sternum. We suggest verifying the identification of this rib by delicately attempting to displace it caudally with surgical tweezers.
- The knot tied to secure the nEMG electrode should be sufficiently tight to prevent its movement but should not be so tight as to impair blood circulation and result in muscle tissue damage.
- Animals should be individually housed for at least 5 days for a complete post-operatory recovery, with daily checks on their health, body weight, and water and food intake. However, Video 8 demonstrates that the mouse appears healthy and exhibits normal behavior the day after surgery.

Figure 13. Implantation of the nuchal electromyographic electrode

- Panels A and B show the insertion of the curved needle on the left and right sides of the neck muscles.
- Panel C shows the final position of the two wires of the nuchal electromyographic electrode secured with a knot.Panel C shows the final position of the two wires of the nuchal electromyographic electrode secured with a knot.Panel C shows the final position of the two wires of the nuchal electromyographic electrode secured with a knot.Panel C shows the final position of the two wires of the nuchal electromyographic electrode secured with a knot.

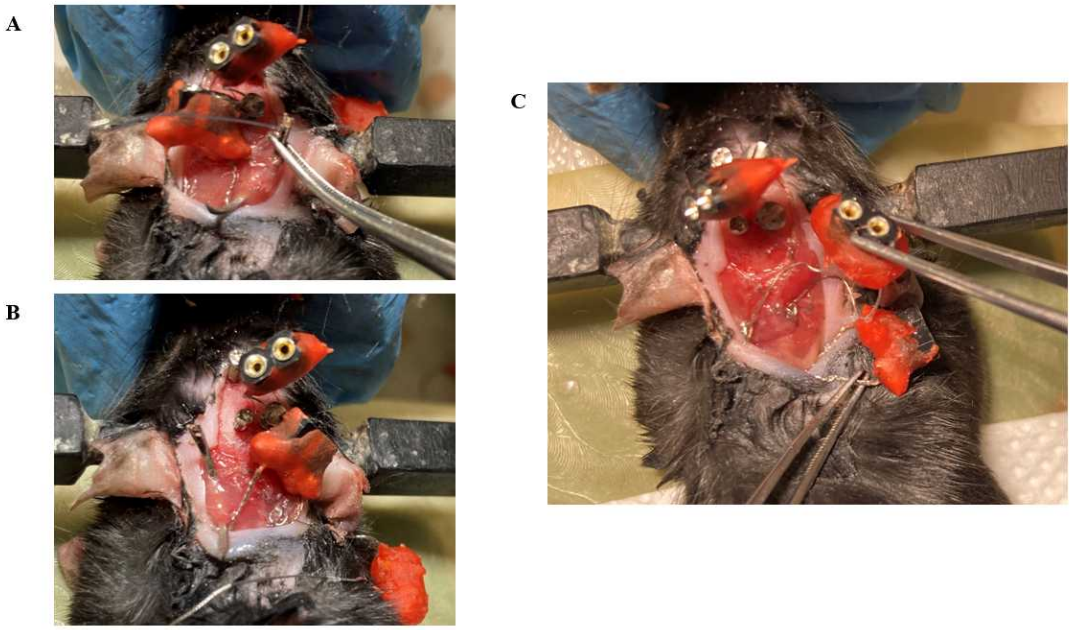

Figure 14. Acrylic structure on mouse's skull

- Photographs of the top (panel A) and side (panel B) view of the mouse with the biocompatible acrylic head cap incorporating the electrodes for electroencephalogram (EEG), nuchal muscle electromyogram (nEMG), and diaphragm electromyogram (DIA) recordings.

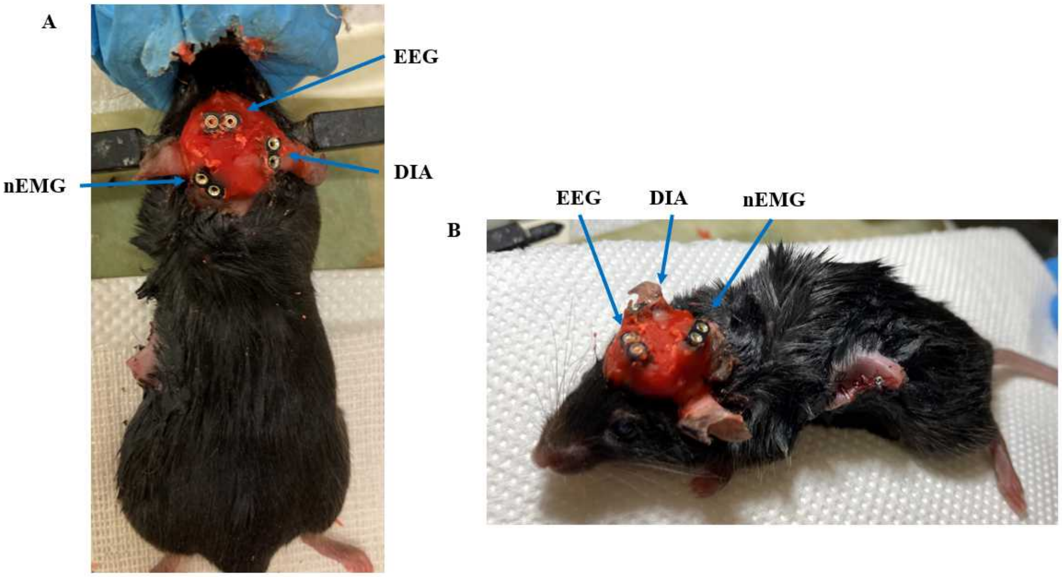

Video 7. Implantation of the electroencephalogram and nuchal muscle electromyogram electrodes

- Detailed step-by-step guide for implanting the electroencephalogram (EEG) and nuchal muscle electromyogram (nEMG) electrodes.

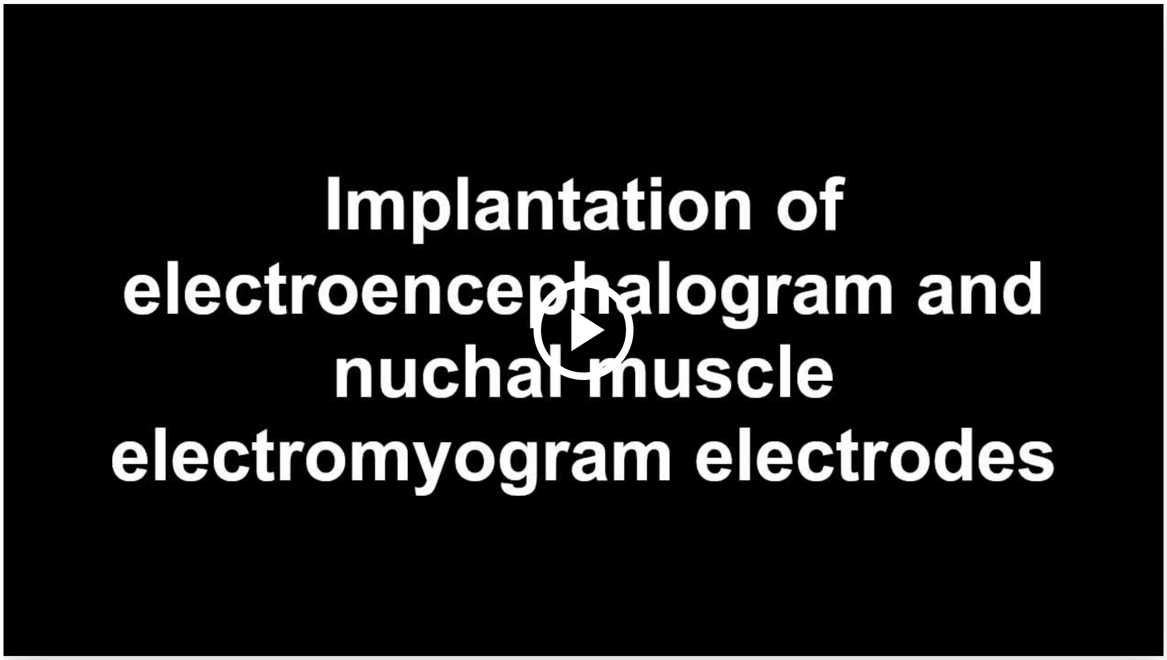

VIDEO 8

Video 8. Mouse recovery

- Typical mouse health condition and behavior the day after the surgery.

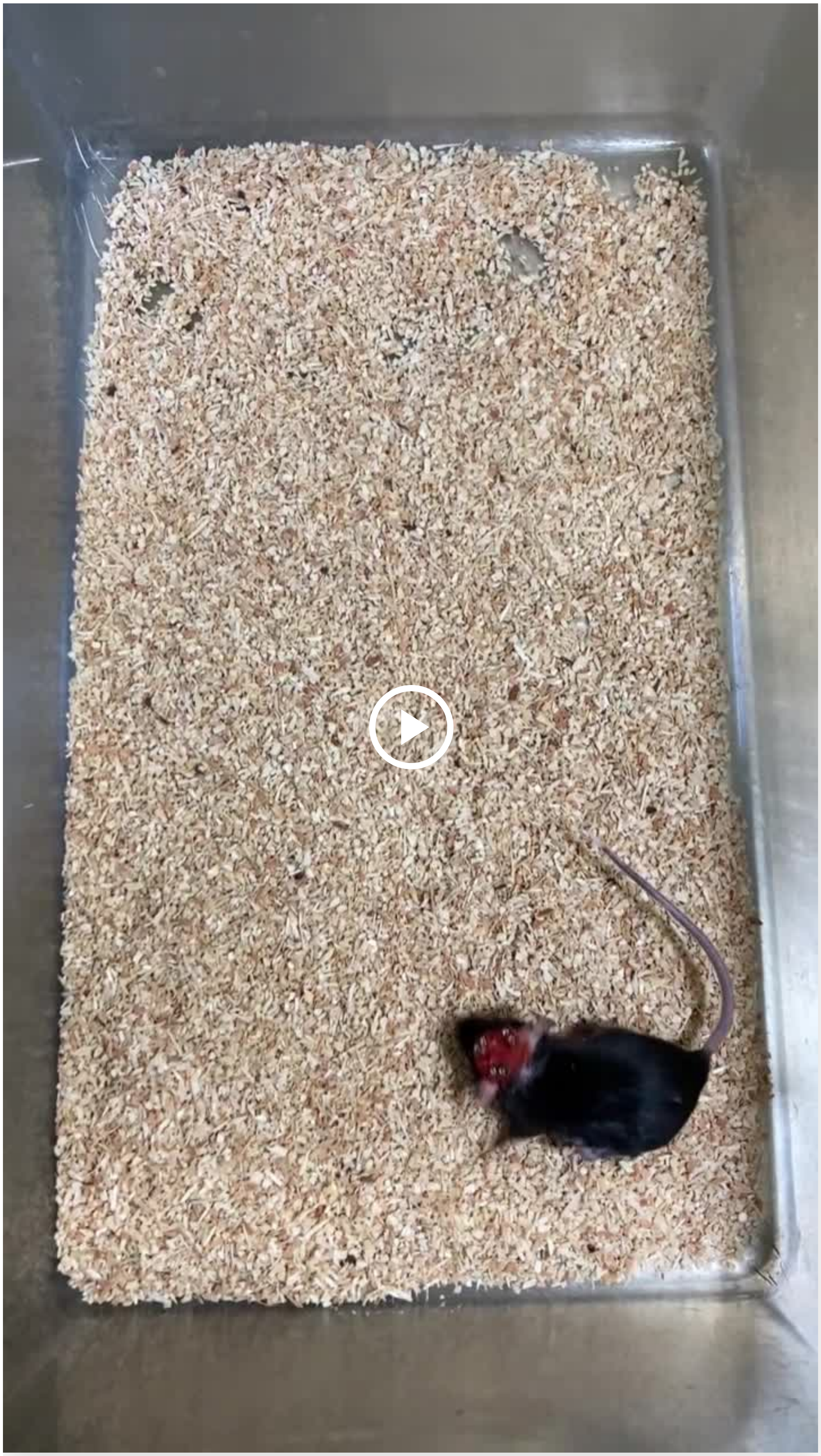

Step 4: Recording within a whole-body plethysmograph

19 In this section, we describe how to prepare and perform the simultaneous recording of the animal's behavioral states, ventilation, and diaphragmatic activity with whole-body plethysmography (WBP).
The WBP apparatus consists of two chambers: one designed for housing the mouse, and a separate reference chamber. Each chamber is equipped with air inlet and outlet tubes. The mouse chamber includes temperature and humidity sensors, and a high-precision differential pressure transducer measures the pressure difference between the two chambers, allowing the detection of the animal's breathing. The mouse body temperature should also be measured ideally.

Note

Timing: 07:00:00 - 08:00:00

20 **Preparation of the animal**

20.1 Before the recording, slightly anesthetize (1–2 minutes under 1-2% isoflurane anesthesia) the mouse to plug a lightweight cable to the electrodes in the head structure for the acquisition of EEG, nEMG, and DIA signals.

21 **Recording within the WBP chamber**

21.1 Insert the mouse into the WBP chamber (Figures 15 and 16). We suggest equipping the apparatus with a rotating electrical commutator to prevent the twisting of the recording cable (Figure 17).

21.2
- The chamber must be continuously purged with a suitable gas mixture to prevent carbon dioxide build-up. The airflow rate should be adjusted according to the size of the plethysmography chamber. For example, our chambers have a 0.97-liter internal volume and are ventilated with breathable air from cylinders at a rate of 1.5 liters per minute.
- The animal respiratory signal is measured as the differential pressure between the chamber containing the animal and a second reference chamber through a high-precision differential pressure transducer, corrected based on the temperature and humidity signals, and calibrated based on the data obtained with the procedure detailed at point 21.3. Mouse body temperature information should also be used to correct tidal volume estimates, if available.
- The EEG, nEMG, and DIA signals are acquired via a lightweight electrical cable, amplified, filtered, digitalized, and stored together with the WBP differential pressure.

21.3 At the end of the session, after removing the mouse from the chamber, calibrate the WBP system by repeatedly injecting and withdrawing air into the mouse chamber with a calibrated micro-syringe while recordings of the differential pressure between WBP chambers are still running.

We recommend sequentially injecting and withdrawing 10, 20, 30, 40, and 50 microliters of air at the anticipated mean breathing rate of the mouse. These data will be used to construct a calibration curve to convert WBP pressure deflection into air volumes.

22 **Figure 15. Whole-body plethysmograph**

- Schematic representation of the modified 2-chamber whole-body plethysmograph, used in our laboratory. The figure shows the Plexiglas block used to reduce the internal volume of the mouse chamber (A), the mouse chamber with the tower equipped with the rotating electrical swivel (B), and the reference chamber (C).

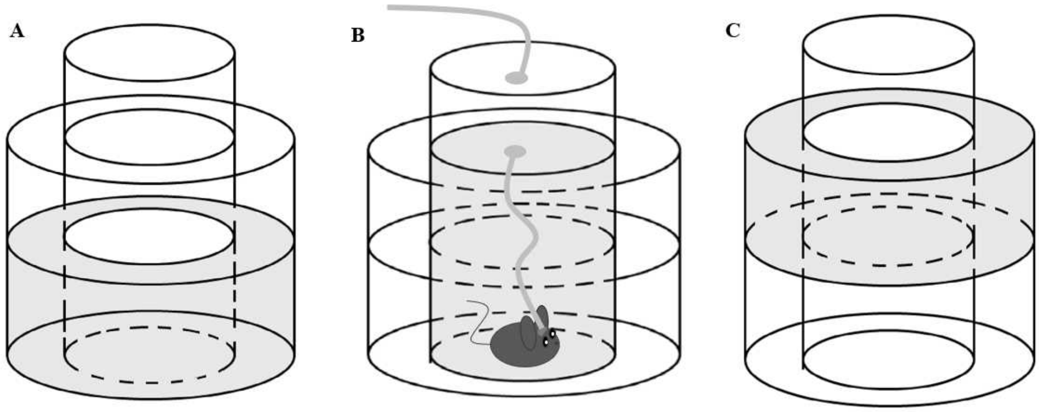

Figure 16. Whole-body plethysmograph

- Photographs of the modified 2-chamber whole-body plethysmograph (panels A and B).

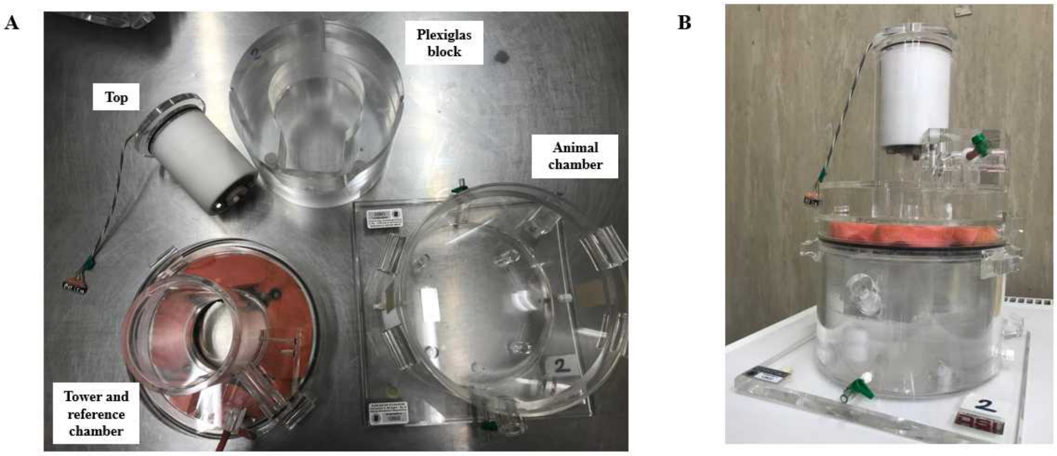

Figure 17. Top of the whole-body plethysmograph

- Panel A shows a schematic representation of the part of the whole-body plethysmograph that was equipped with a rotating electrical commutator (blue-lined segment and cylinders) that prevented the twisting of the recording lightweight cable (green segment) connected to the electrodes included in the acrylic cap on the mouse's head (red semicircle).
- Panel B shows photographs of the top of the whole-body plethysmograph equipped with the rotating electrical commutator.

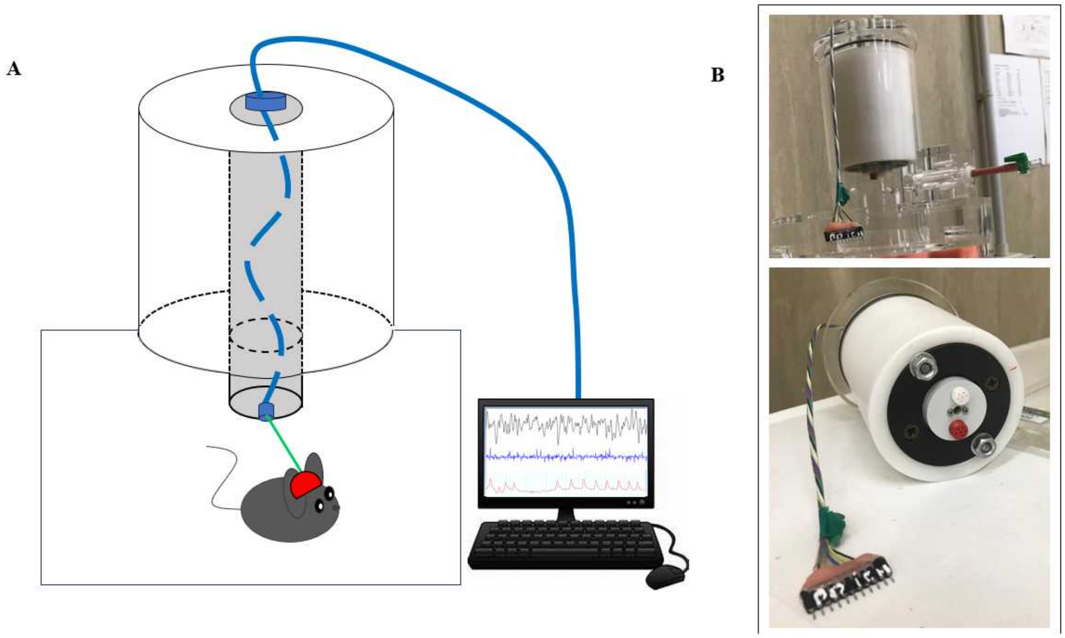

Note

Tips:

- We suggest filtering the signals as follows: EEG 0.3–100 Hz; nEMG and DIA 100–1000 Hz.
- We sample the signals at the following rates: EEG, 128 Hz; nEMG, DIA, and WBP differential pressure, 2048 Hz.
- In case of significant electrical artifacts due to alternating current (50 or 60 Hz, according to the country) in one of the differential signals, we suggest attempting an alternative derivation for the signal. For example, if a significant electrical artifact occurs in the differential signal between the two pins of the EEG electrode, consider acquiring a differential signal between one EEG electrode pin and one nEMG electrode pin as reference. Please, note that, while this new configuration may still permit accurate sleep scoring, it is not suitable for EEG power spectral analysis.
- To minimize mechanical artifacts in the WBP differential pressure signal caused by the opening and closing of doors, we recommend limiting the entrance and exit in the recording laboratory to the essential minimum.

Note

Critical points:

- Remember to measure the temperature and the humidity of the animal chamber, and ideally also the animal's body temperature, as they are relevant to the analysis of the WBP signal (1).

CITATION

Drorbaugh J.E., Fenn W.O. (1955). A barometric method for measuring ventilation in newborn infants. Pediatrics.

LINK

1955 Jul;16(1):81-7.

- Always verify the proper sealing of the WBP chamber to ensure the acquisition of a high-quality differential pressure signal.
- WBP chambers are generally employed for recordings lasting 6-8 hours; with longer continuous recordings, waste (feces, urine, wet food pellets) in the mouse chamber may impact on the mouse well-being and behavior.

Step 5: Discrimination of wake-sleep states in mice

23 In this section, we describe how to discriminate the different behavioral states in mice based on the EEG and nEMG signal traces according to published criteria.
A more detailed description with figures and a specific MATLAB script for wake-sleep state discrimination in mice can be found in *Bastianini et al., 2014* (2).

CITATION

Bastianini S, Berteotti C, Gabrielli A, Del Vecchio F, Amici R, Alexandre C, Scammell TE, Gazea M, Kimura M, Lo Martire V, Silvani A, Zoccoli G (2014). SCOPRISM: a new algorithm for automatic sleep scoring in mice..
LINK
https://doi.org/10.1016/j.jneumeth.2014.07.018

We recommend scoring mouse sleep with a 4-second time resolution.

Note

Timing: variable (expert investigators can score a 7-hour recording with a 4-second time resolution in about 01:00:00)

24 **Perform the sleep scoring of the entire recording by visual inspection of raw EEG and nEMG signals (2–4) with the following criteria:**

- Wakefulness (W) is assigned when the nEMG tone is high and variable, and the EEG is at a low voltage with possible δ (0.5 – 4 Hz) and θ (6 – 9 Hz) frequency components.
- Non-rapid-eye movement sleep (NREMS) is scored when the nEMG tone is lower than in W, and the EEG is at a high voltage with prominent δ frequency components.
- Rapid-eye movement sleep (REMS) is defined in the presence of muscle atonia (flat nEMG) with occasional muscle twitches, and when the EEG is at a low voltage with predominant θ activity.
- Epochs with artifacts or borderline signals should be marked as “undetermined” and excluded from the successive analyses.

CITATION

Bastianini S, Berteotti C, Gabrielli A, Del Vecchio F, Amici R, Alexandre C, Scammell TE, Gazea M, Kimura M, Lo Martire V, Silvani A, Zoccoli G (2014). SCOPRISM: a new algorithm for automatic sleep scoring in mice..
LINK
https://doi.org/10.1016/j.jneumeth.2014.07.018

CITATION

Franken P., Malafosse A., Tafti M. (1999). Genetic determinants of sleep regulation in inbred mice. Sleep.
LINK
1999 Mar 15;22(2):155-69.

CITATION

Silvani A, Bastianini S, Berteotti C, Franzini C, Lenzi P, Lo Martire V, Zoccoli G (2009). Sleep modulates hypertension in leptin-deficient obese mice..
LINK
https://doi.org/10.1161/HYPERTENSIONAHA.108.125542

25 **Figure 18. Discrimination of the wake-sleep states**

- Example of raw electroencephalographic (EEG) and nuchal electromyographic (nEMG) data obtained during wakefulness (W), non-rapid-eye-movements sleep (NREMS), and rapid-eye-movements sleep (REMS) in mice in our laboratory.

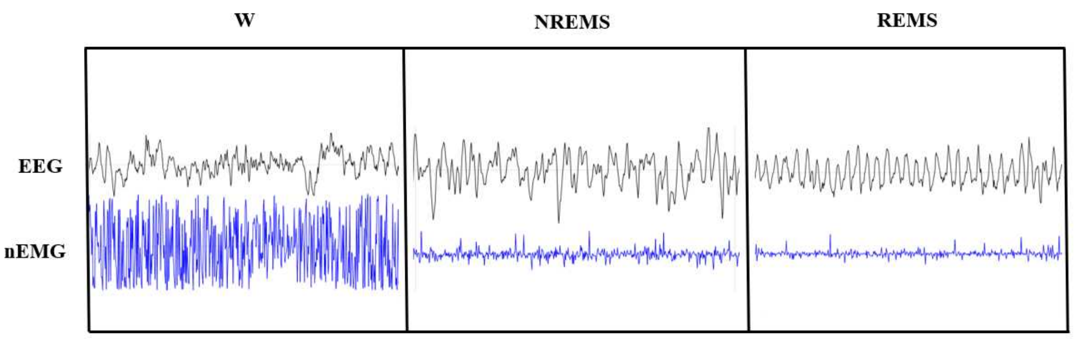

Step 6: Sleep apnea detection and categorization into CSA and OSA

26 In this section, we first describe how to detect sleep apnea from the WBP signal and then, how to categorize sleep apneas into CSA and OSA based on the joint evaluation of the WBP and DIA signals.

Note

Timing: variable (depending on the number of apneas to be evaluated; the examination of a 7-hour WBP recording typically takes about 01:00:00)

27 **Sleep apnea detection**

27.1 Using commercially available or custom-made algorithms, calculate the ventilatory period (VP, i.e., the duration of each breath) on the raw WBP tracings, computed as the time period between two consecutive peaks of the WBP signals.

27.2 Calculate the mean VP value for each mouse in each sleep state (i.e., NREMS and REMS).

27.3 Calculate the apnea threshold for each sleep state, setting it at either two times (2x criterion) or three times (3x criterion, which is the more conservative criterion used in our lab) the mean VP values defined for each mouse.

27.4 Using commercially available or custom-made algorithms, for each sleep state, detect all the breaths longer than the apnea threshold and discard false detections due to artifacts (e.g., due to high-amplitude mechanical pressure artifacts caused by the opening or closing of laboratory doors) by visual evaluation of the raw tracings.

28 Figure 19 shows a representative tracing of the WBP differential pressure signal recorded inside the WBP chamber in our laboratory.

Figure 19. Ventilatory period

- The figure shows a representative tracing of the whole-body plethysmography differential pressure signal (in red) recorded in mice in our laboratory. Grey segments identify the ventilatory period (VP, i.e., the duration of each breath) associated with an apnea (red bracket) and some normal breaths (green bracket). The time scale is one second.

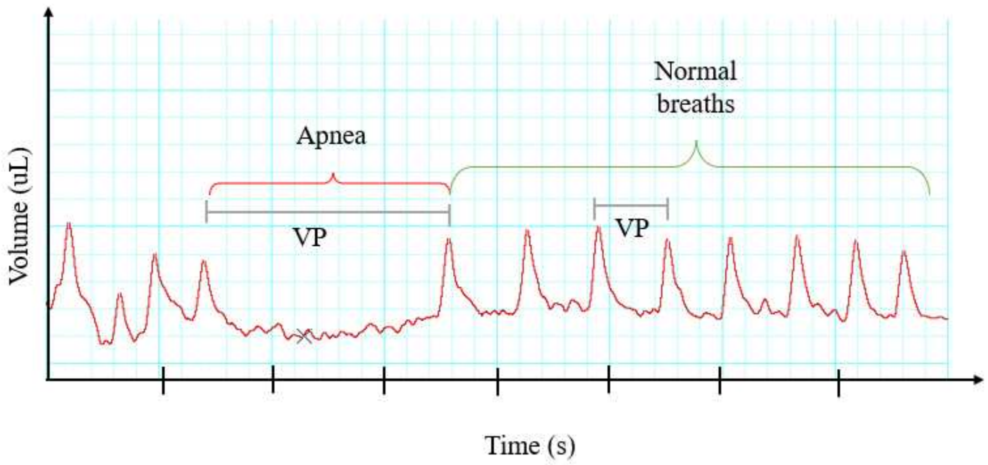

Note

Tips:

- We suggest confining the analysis of respiratory variables to stable periods of NREMS and REMS lasting at least 12 seconds. The analysis of respiratory variables during wakefulness is usually particularly problematic in terms of mechanical artifacts in the WBP pressure signal.
- For a more conservative analysis of apneas, we suggest using the 3x criterion as the apnea threshold; for more details, please refer to Bastianini et al., 2019 (5).

CITATION

Bastianini S, Alvente S, Berteotti C, Bosi M, Lo Martire V, Silvani A, Valli A, Zoccoli G (2019). Post-sigh sleep apneas in mice: Systematic review and data-driven definition..

LINK

https://doi.org/10.1111/jsr.12845

29 **Categorization into CSA and OSA of each manually checked apnea episode**

29.1 Classify the apnea as CSA when the absence of activity in WBP is concomitant with the absence of activity in the DIA signal.

29.2 Classify the apnea as OSA when the absence of activity in WBP is coupled to one or more activity bursts on the DIA signal (i.e., with a clear activation of the diaphragm).

30 Figures 20 and 21 show representative examples of CSA and OSA events recorded inside the WBP chamber in our laboratory.

Figure 20. Central sleep apneas

- Representative examples of central sleep apneas (CSA) recorded inside a whole-body plethysmography chamber in mice in our laboratory. The whole-body plethysmography differential pressure signal (VENT) is represented in red, while the diaphragmatic electrical activity (DIA) is in blue. Green brackets identify CSA events and blue brackets show the lack of contraction of the diaphragm during the apnea.

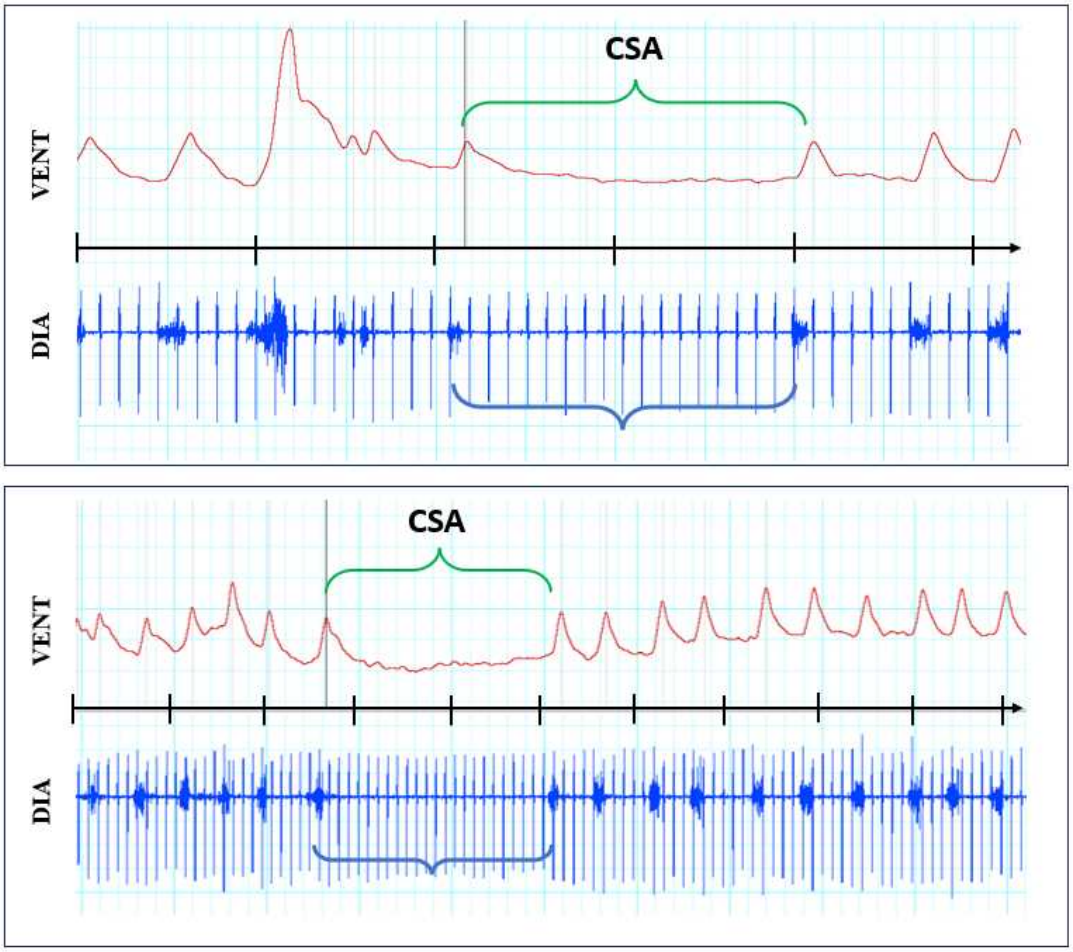

Figure 21. Obstructive sleep apneas

- Representative examples of obstructive sleep apneas (OSA) recorded inside a whole-body plethysmography chamber in mice in our laboratory. The whole-body plethysmography differential pressure signal (VENT) is represented in red, while the diaphragmatic electrical activity (DIA) is in blue. Red brackets identify OSA events and blue circles show the contraction of the diaphragm during the apnea.

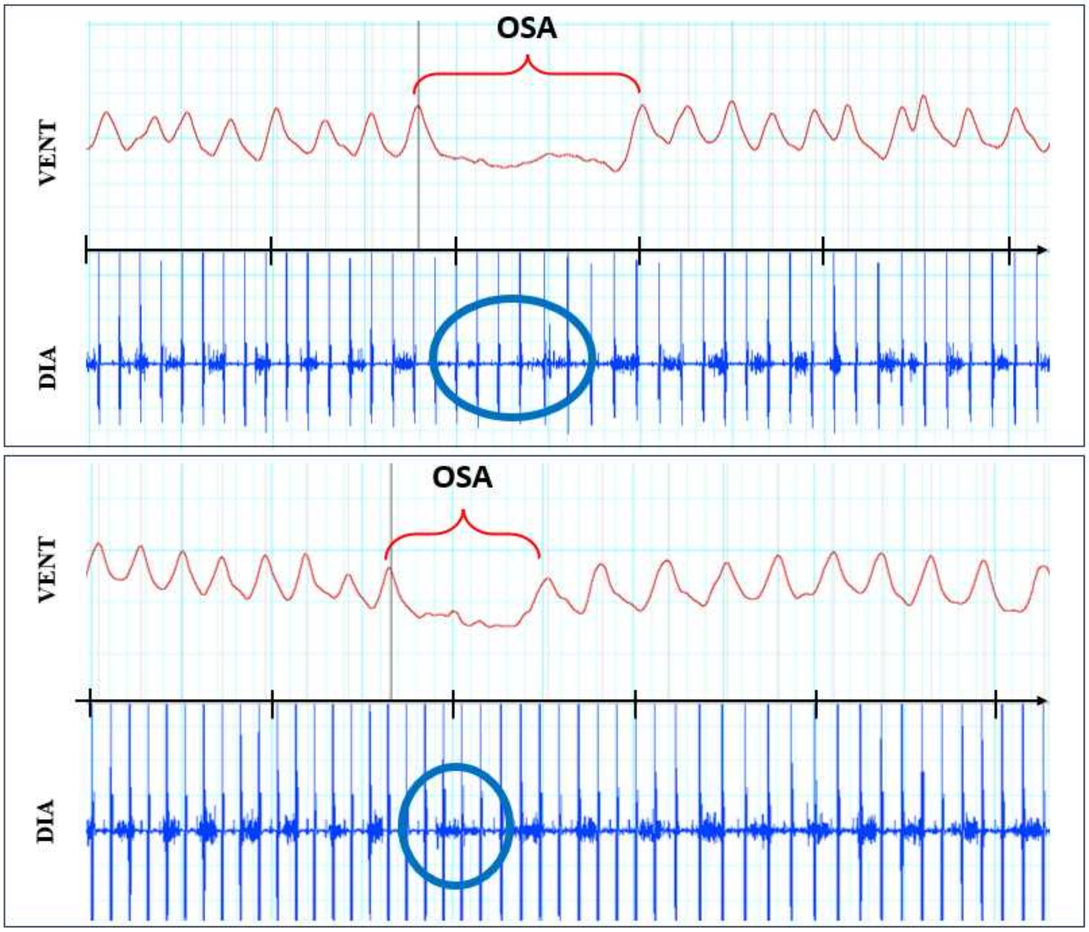

Note

Critical points:

- If you are interested in recording rare events occurring during sleep, we recommend conducting multiple recordings of the same animals.

Protocol references

1. Drorbaugh JE, Fenn WO. A barometric method for measuring ventilation in newborn infants. Pediatrics. 1955 Jul;16(1):81–7.
2. Bastianini S, Berteotti C, Gabrielli A, Del Vecchio F, Amici R, Alexandre C, et al. SCOPRISM: a new algorithm for automatic sleep scoring in mice. J Neurosci Methods. 2014 Sep 30;235:277–84.
3. Franken P, Malafosse A, Tafti M. Genetic determinants of sleep regulation in inbred mice. Sleep. 1999 Mar 15;22(2):155–69.
4. Silvani A, Bastianini S, Berteotti C, Franzini C, Lenzi P, Lo Martire V, et al. Sleep modulates hypertension in leptin-deficient obese mice. Hypertension. 2009 Feb;53(2):251–5.
5. Bastianini S, Alvente S, Berteotti C, Bosi M, Martire VL, Silvani A, et al. Post-sigh sleep apneas in mice: Systematic review and data-driven definition. Journal of sleep research [Internet]. 2019 Dec;28(6).

Citations

Bastianini S, Berteotti C, Gabrielli A, Del Vecchio F, Amici R, Alexandre C, Scammell TE, Gazea M, Kimura M, Lo Martire V, Silvani A, Zoccoli G. SCOPRISM: a new algorithm for automatic sleep scoring in mice.
https://doi.org/10.1016/j.jneumeth.2014.07.018

Step 22

Drorbaugh J.E., Fenn W.O.. A barometric method for measuring ventilation in newborn infants
1955 Jul;16(1):81-7.

Step 23

Bastianini S, Berteotti C, Gabrielli A, Del Vecchio F, Amici R, Alexandre C, Scammell TE, Gazea M, Kimura M, Lo Martire V, Silvani A, Zoccoli G. SCOPRISM: a new algorithm for automatic sleep scoring in mice.
https://doi.org/10.1016/j.jneumeth.2014.07.018

Step 24

Bastianini S, Berteotti C, Gabrielli A, Del Vecchio F, Amici R, Alexandre C, Scammell TE, Gazea M, Kimura M, Lo Martire V, Silvani A, Zoccoli G. SCOPRISM: a new algorithm for automatic sleep scoring in mice.
https://doi.org/10.1016/j.jneumeth.2014.07.018

Step 24

Franken P., Malafosse A., Tafti M.. Genetic determinants of sleep regulation in inbred mice
1999 Mar 15;22(2):155-69.

Step 24

Silvani A, Bastianini S, Berteotti C, Franzini C, Lenzi P, Lo Martire V, Zoccoli G. Sleep modulates hypertension in leptin-deficient obese mice.
https://doi.org/10.1161/HYPERTENSIONAHA.108.125542

Step 28

Bastianini S, Alvente S, Berteotti C, Bosi M, Lo Martire V, Silvani A, Valli A, Zoccoli G. Post-sigh sleep apneas in mice: Systematic review and data-driven definition.
https://doi.org/10.1111/jsr.12845
