# Supplementary material for: Detection of central and obstructive sleep apneas in mice: A new surgical and recording protocol
Source: PLoS One. 2025 Mar 28;20(3):e0320650. doi: 10.1371/journal.pone.0320650 (PMC12048029; doi:10.1371/journal.pone.0320650)
Supplement: Attachment — Submitted filename: Response to Reviewers.docx [file pone.0320650.s003.docx]

**Rebuttal letter to the editor and reviewers**

**We sincerely appreciate the two Reviewers for acknowledging the significance of our study and providing valuable feedback. We have revised the manuscript following their suggestions to enhance the clarity and strength of our scientific message.**

**Below, we provide a detailed response to each of the points raised. All revisions are highlighted in red in the file titled "Revised Manuscript with Track Changes."**

**Reviewer #1:**

Dear Authors,

I read the manuscript entitled: Detection of central and obstructive sleep apneas in mice: a new surgical and recording protocol. In this manuscript authors described a method that can identify central and obstructive sleep apneas in mice. This method involves the surgical implantation of electrodes for recording the electroencephalogram and nuchal muscle electromyogram, which are the gold standard to study the wake-sleep cycle, and for recording the diaphragm electromyogram, which allows the detection of diaphragm contraction. This method also allows to score wake-sleep states and to detect sleep apneas and categorize them into central and obstructive events.

Animal models are crucial tools in biomedical and preclinical research, enabling reliable assessment of the safety and effectiveness of new therapies before they proceed to human trials. In the case of Obstructive Sleep Apnea (OSA), they are particularly valuable for advancing our understanding of its pathophysiology.

1. The introduction of the article is brief and concise. I believe, it is important to emphasize in the introduction the significance of this protocol by comparing it with what is already established in the literature.
2. There are several important studies exploring Obstructive Sleep Apnea (OSA) in mice, particularly focused on how intermittent hypoxia (IH), a hallmark of OSA, affects various physiological systems. This protocol could significantly advance research on Obstructive Sleep Apnea (OSA) by addressing gaps or limitations present in current literature.

**We have expanded the “Introduction” section to emphasize the significance of intermittent hypoxia protocols in investigating the pathophysiological effects of obstructive sleep apnea in mice (lines 82-93). We also provided a brief overview of current methods employed to distinguish between CSA and OSA in mice, with a specific focus on the technique developed by Polotsky and colleagues (lines 94-99) and the approach employed in our laboratory (lines 100-108). These methods are further discussed in the “Expected results” section of the present paper.** **Finally, the importance of our method, which can be seamlessly integrated with existing intermittent hypoxia protocols, is also highlighted in the “Conclusions” section (lines 233-236). To address these requests, we have included the following citations: *Farrè et al., 2003*; *Almendros et al., 2011*; *Schoorlemmer et al., 2011*; *Lebek et al., 2020*; *Allaband et al., 2021*; *Hu et al., 2021*.**

**Reviewer #2**:

Dear Authors,

I read your manuscript with great interest since, as authors pointed out, the article deals with a topic that is -still little known. I found very attractive your protocol and I'm very sure that it will be appreciated by the readers. Furthermore the manuscript is well written and the results are very interesting. Wishing you the best in your work.

**We sincerely thank the Reviewer for the kind compliments. We truly appreciate his/her positive feedback on our manuscript and are grateful that he/she found our protocol interesting and valuable.**

**Editor for Journal requirements**:

1. Please ensure that your manuscript meets PLOS ONE's style requirements, including those for file naming.

**We reviewed and modified the manuscript according to the “Manuscript body formatting guidelines” and the “Title, author, affiliation formatting guidelines”.**

2. To comply with PLOS ONE submissions requirements, in your Methods section, please provide additional information regarding the experiments involving animals and ensure you have included details on (1) methods of sacrifice, (2) methods of anesthesia and/or analgesia, and (3) efforts to alleviate suffering.

**In addition to the information on anesthesia and the efforts to minimize the suffering of the experimental animals already included in the manuscript (lines 118-123), we have expanded the "Materials and Methods" section to provide further details on the anesthesia and analgesia protocols used during surgery (lines 125-131) as well as the methods of euthanasia (lines 133-137).**

3. Thank you for stating the following financial disclosure: “This work was co-funded by the European Union (NextGenerationEU) under the National Recovery and Resilience Plan (PNRR) within the framework of the project “ESTROSA: Energy-autonomous System for TReatment of Obstructive Sleep Apnea” (Code MUR P2022RTCCA_001 – CUP J53D23014050001) and by the Italian Ministry of Health with the grant PRIN2022 for the project “CORSA: Chemogenetic and Optogenetic Rescue of Sleep Apnea in mice” (Code MUR 2022CR32TM_001 – CUP J53D2301094000) awarded to S.B.”

Please state what role the funders took in the study. If the funders had no role, please state: "The funders had no role in study design, data collection and analysis, decision to publish, or preparation of the manuscript." If this statement is not correct you must amend it as needed. Please include this amended Role of Funder statement in your cover letter; we will change the online submission form on your behalf.

**The funders were not involved in the study design, data collection and analysis, decision to publish, or manuscript preparation. This statement has been included in the “Financial Disclosure statement” section (lines 23-24) and in the revised cover letter.**

4. Your ethics statement should only appear in the Methods section of your manuscript. If your ethics statement is written in any section besides the Methods, please move it to the Methods section and delete it from any other section. Please ensure that your ethics statement is included in your manuscript, as the ethics statement entered into the online submission form will not be published alongside your manuscript.

**Our ethics statement is included in the "Materials and Methods" section of the manuscript (lines 118-123).**

5. Please review your reference list to ensure that it is complete and correct. If you have cited papers that have been retracted, please include the rationale for doing so in the manuscript text, or remove these references and replace them with relevant current references. Any changes to the reference list should be mentioned in the rebuttal letter that accompanies your revised manuscript. If you need to cite a retracted article, indicate the article’s retracted status in the References list and also include a citation and full reference for the retraction notice.

**We have reviewed our reference list and added the following citations to address the requests raised by the reviewers: *Farrè et al., 2003*; *Almendros et al., 2011*; *Schoorlemmer et al., 2011*; *Lebek et al., 2020*; *Allaband et al., 2021*; *Hu et al., 2021*.**
